# Microrobotic tentacles with spiral bending capability based on shape-engineered elastomeric microtubes

**DOI:** 10.1038/srep10768

**Published:** 2015-06-11

**Authors:** Jungwook Paek, Inho Cho, Jaeyoun Kim

**Affiliations:** 1 Department of Electrical and Computer Engineering Iowa State University, Ames, Iowa, USA; 2 Department of Civil, Construction and Environmental Engineering Iowa State University, Ames, Iowa, USA

## Abstract

Microscale soft-robots hold great promise as safe handlers of delicate micro-objects but their wider adoption requires micro-actuators with greater efficiency and ease-of-fabrication. Here we present an elastomeric microtube-based pneumatic actuator that can be extended into a microrobotic tentacle. We establish a new, direct peeling-based technique for building long and thin, highly deformable microtubes and a semi-analytical model for their shape-engineering. Using them in combination, we amplify the microtube’s pneumatically-driven bending into multi-turn inward spiraling. The resulting micro-tentacle exhibit spiraling with the final radius as small as ~185 μm and grabbing force of ~0.78 mN, rendering itself ideal for non-damaging manipulation of soft, fragile micro-objects. This spiraling tentacle-based grabbing modality, the direct peeling-enabled elastomeric microtube fabrication technique, and the concept of microtube shape-engineering are all unprecedented and will enrich the field of soft-robotics.

Elastomer-based soft-robots are gaining popularity as safe handlers of delicate objects^1^,^2^,^3^,^4^,^5^,^6^ For applications like *in vivo* biomedical manipulation^7^,^8^,^9^,^10^, efforts have been underway for their microscale miniaturization as well^10^,^11^,^12^,^13^,^14^,^15^,^16^,^17^ but finding efficient actuators for microscale soft-robots remains a difficult task^9^,^18^,^19^,^20^,^21^,^22^,^23^,^24^,^25^ Pneumatic actuation is a good candidate with its simplicity and efficiency already proven in large-scale soft-robotics^5^,^26^,^27^ but its microscale implementation entails many technical challenges^28^,^29^ First, current soft-lithographic microfabrication techniques, developed mainly for building planar elastomer structures with low aspect-ratio patterns such as microchannels, are not optimal for constructing three-dimensional, hermetically sealed cavities required for pneumatic actuation. We can still build them by bonding two planar structures^11^,^12^,^13^,^14^,^30^ or employing dissolvable templates^31^ But the strength and yield of bonding decrease with the length-scale. Dissolving templates often becomes an equally complex task at microscale. Moreover, large-scale pneumatic actuators rely on many sub-elements, such as surface corrugation or valves^28^,^29^, that are neither simple nor desirable to replicate exactly at microscale. In all, a successful microscale realization of a pneumatic soft-actuator requires a combination of new designs and fabrication techniques optimized specifically to that length-scale.

Here, we present one such combination for realizing a microtube-type pneumatic soft-actuator that can be extended to microrobotic tentacles. Spiraling tentacles are widely utilized in nature for grabbing and squeezing objects. There have been continuous soft-robotic efforts to mimic them with pneumatic tube actuators^32^,^33^ but the life-like, multi-turn spiraling motion has been reproduced only by centimeter-scale tentacles so far^5^,^34^ At millimeter- and sub-millimeter scales, they could bend only up to a single-turn^12^,^14^,^31^,^35^ Since the bending arises from the mismatch in the elongation levels of the tube’s top and bottom sides, it can be amplified into spiraling through mismatch enhancement. At macroscale, it is typically done with bi-elastomeric composite structures^5^ or highly modulated surface corrugations^34^ Neither is, however, easy to implement at microscale.

Our approach is two-stepped. In the first, we fabricate easily deformable elastomeric microtubes as the platform structure. High deformability is ensured by the thinness of the microtube itself (100−125 μm in inner diameter) and its tube-wall (8−32 μm). Making such a subtle structure at a length exceeding several millimeters has been deemed unfeasible. We accomplish it with a new, direct peeling-based soft-lithographic technique. It also allows significant asymmetrization of the microtube’s cross-sectional shape which leads to bending up to a single-turn. In the second step, we apply shape-engineering to the microtube to amplify the bending into multi-turn spiraling. Using a semi-analytical model, we establish a design rule which enables such a spiraling with a simple hump (Fig. 1a). The outcome is a soft-robotic micro-tentacle that can wind around and hold fragile micro-objects with ~200 μm final spiral radius. This spiraling micro-tentacle manipulator, along with the shape-engineering concept and microtube fabrication technique, are all unprecedented and poised to enrich the field of soft-robotics.

## Results

### Peeling-based fabrication of PDMS microtubes

**Figure 1:**
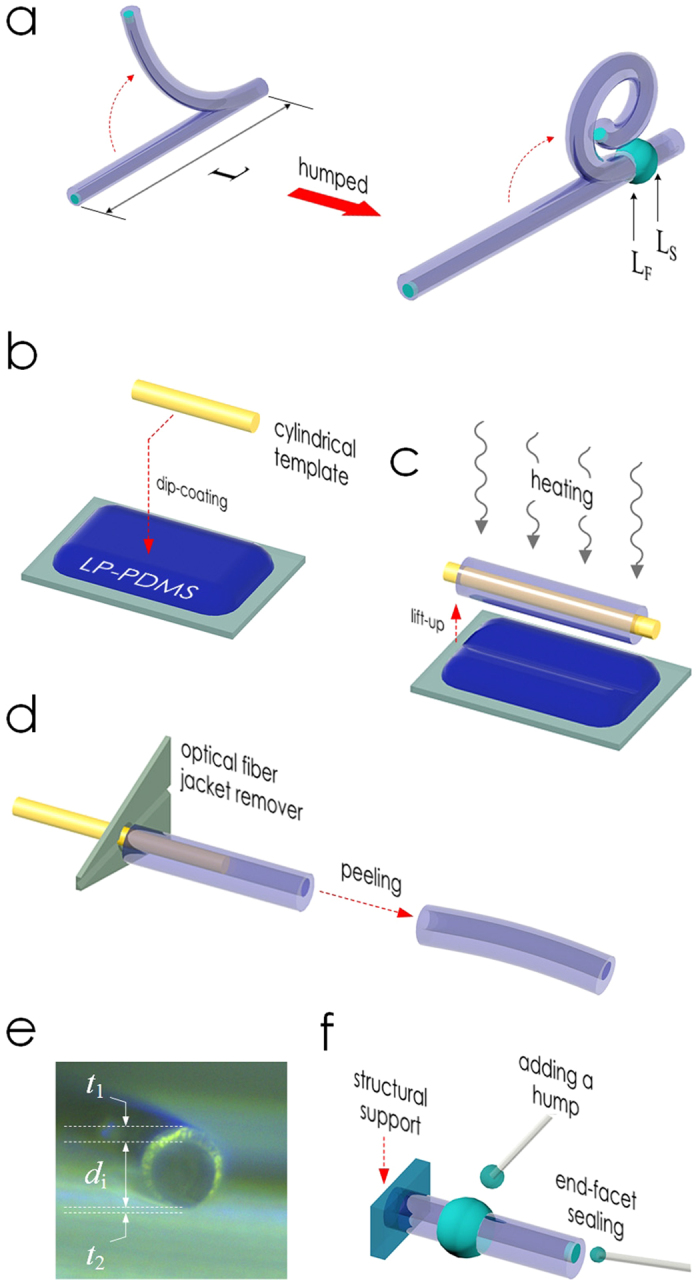
PDMS microtube tentacle actuator and its fabrication. (**a**) A schematic diagram of PDMS microtube tentacle actuator. Unlike the plain microtube (left), the one shape-engineered with a hump (right) can produce a tentacle-like spiraling motion. Fabrication steps: (**b**) Dip-coating of a cylindrical template with liquid-phase (LP) PDMS, (**c**) Lifting up of the PDMS-coated template with *in situ* heating, (**d**) Peeling of the cured PDMS microtube with a fiber-optic jacket remover, (**e**) The cross-sectional optical micrograph of a microtube shows the gravity-induced asymmetry (*t*_1_ ≠ *t*_2_). (**f**) Additional structures, such as the mount, end-facet sealing, and hump, can be installed to the already completed and tested microtube.

Past reports testify that the biggest issue in using liquid-phase poly(dimethylsiloxane) (PDMS) for building long and thin structures, such as pillars or wires, is its tendency to bead^36^ In our previous work, we fabricated ultra-high aspect-ratio PDMS micropillars by suppressing the beading with pre-curing and *in situ* thermal solidification^37^ Here, we extend the technique to liquid-phase PDMS dip-coated around cylindrical templates to realize PDMS microtubes with very thin walls without bonding (Fig. 1b,c).

The completed PDMS microtube is robust enough to be peeled directly from the template with a polymer jacket remover, a standard tool in fiber-optics (Figs. 1d). The process is simpler, cleaner, and faster than those requiring dissolvable templates^38^,^39^ Using polyamide wires (~100 μm diameter) and glass optical fibers (125 μm diameter) as the template, we fabricated microtubes with their length *L* reaching 5−8 mm. The main factor limiting *L* was the inevitable increase in the friction and wrinkling during the peeling process. Despite their high aspect-ratios (typically > 50) and thin walls, the microtubes did not sag or collapse, even when no air pressure was applied.

The solidification process can also be controlled for additional tailoring of the microtube’s cross-sectional shape. Slowing it down prolongs gravitational impact on the coating, resulting in an increase in the tube’s cross-sectional asymmetry, as shown in Fig. 1e. The 10 samples we fabricated with the 100 μm-diameter template exhibited *t*_1_ and *t*_2_ of 31.8 ± 4.9 (s.d.) and 7.9 ± 1 (s.d.) μm, respectively. The coating thickness can also be made uniform by rotating the template during curing.

As shown in Fig. 1f, we can also mount the microtube on another PDMS block or modify its shape. Closing the open end with a drop of liquid-phase PDMS for hermetic sealing is a common procedure. The same drop can be dispensed at any point along the microtube to form a monolithically integrated hump. We exploited this capability extensively to shape-engineer the microtube.

### Pneumatic actuation of PDMS microtubes

**Figure 2:**
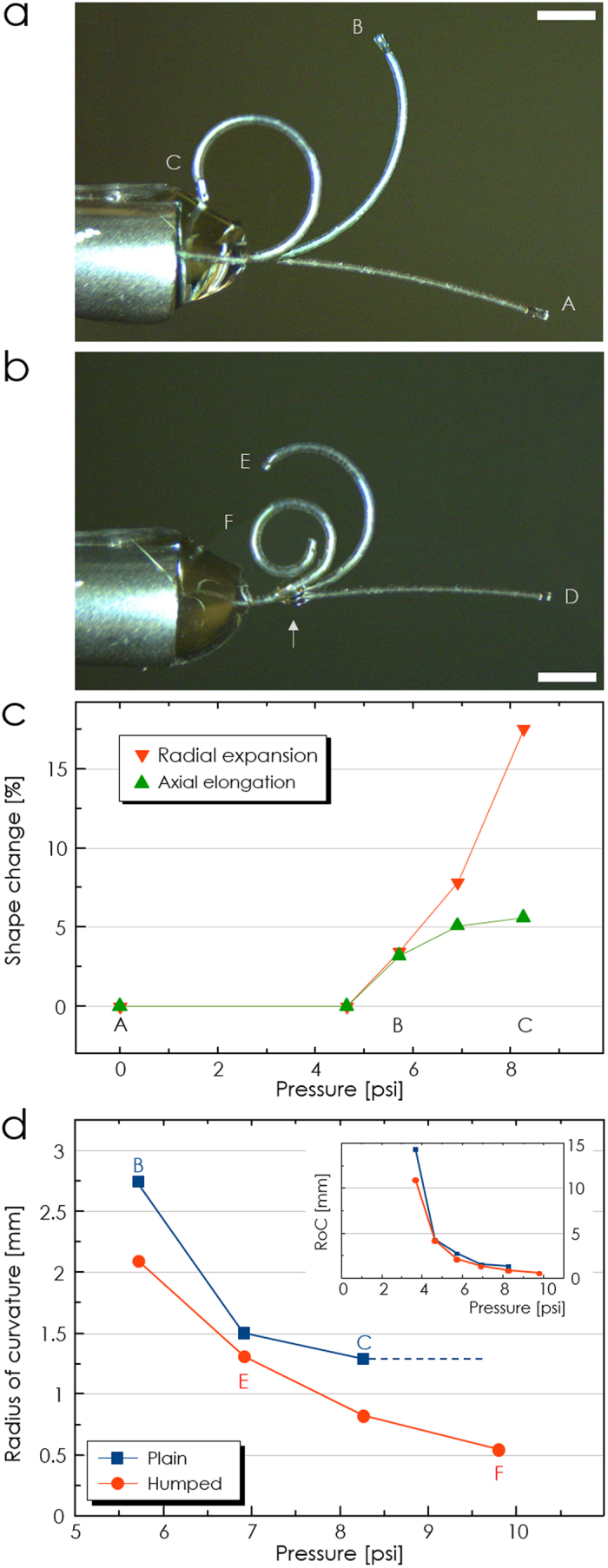
Pneumatic actuation of PDMS microtubes. Superimposed images of pneumatically actuated PDMS microtube MT1. Labels A-F indicate pressure levels marked in Fig. 2c and d. The cylinder on the left-hand side is a blunt syringe needle with 2.108 mm outer diameter. (**a**) Before adding a hump, its bending did not develop into spiraling. (**b**) Adding a hump at the arrow point amplified MT1’s bending into spiraling. (Scale bars: 1 mm) (**c**) Axial and radial shape changes observed in MT1 (before adding the hump) as a function of pressure. (**d**) Changes in MT1’s RoC. Without the hump, it saturated at ~1.3 mm. The hump reduced it to ~500 μm. The inset shows that the RoC decreased rapidly once the pressure surpassed the threshold value at ~4.7 psi.

We connected the microtubes directly to blunt syringe needles for pneumatic actuation. Owing to their high aspect-ratio, thin tube-wall, and inherent softness of PDMS, the microtubes bent significantly upon applying air pressure, as shown in Fig. 2a,b.

As the characterization sample, we used the one in Fig. 2a (MT1) with *L*, *d*_i_, *t*_1_, and *t*_2_ at 5600, 104, 35.6, and 7.7 μm, respectively. Figure 2c shows the levels of its axial elongation and radial expansion at different pressure levels. Clearly, the two morphological changes occurred with a common threshold in the applied pressure (~4.7 psi) below which they became negligible. Beyond the threshold, both the length and radius changed rapidly, eventually getting increased by 5% and 18%, respectively. Such large deformations caused the cross-sectionally asymmetric microtube to bend. Figure 2d and its inset show the decrease in MT1’s radius of curvature (RoC) which also changed abruptly as the pressure surpassed the threshold value. The RoC of MT1 saturated at 1.3 mm at 8.3 psi, forming a ring as shown in Fig. 2a. Other microtubes exhibited similar responses.

Regardless of the pressure or cross-sectional asymmetry, plain microtubes with no cross-sectional change along in the axial direction failed to achieve spiraling. Conventionally, such an insufficient bending has been amplified into spiraling through the use of bi-material composite structures^5^ or bellows-like surface corrugations^34^,^40^ which promote the mismatch in the elongation capabilities. They are, however, very complex to implement at microscale.

### Shape-engineering for tentacle motion

Instead, we enabled the spiraling motion in the PDMS microtube actuator by adding a simple hump to it as shown in Figs. 1f and 2b. In terms of fabrication, this approach is highly advantageous since it requires access only to the microtube’s exterior and, hence, can be executed after the microtube is completely fabricated and tested. Figure 2b shows the change in MT1’s pneumatic actuation after a 610 μm-long, 96 μm-high hump was added. It clearly exhibited spiraling with the minimum RoC reduced to 500 μm.

**Figure 3:**
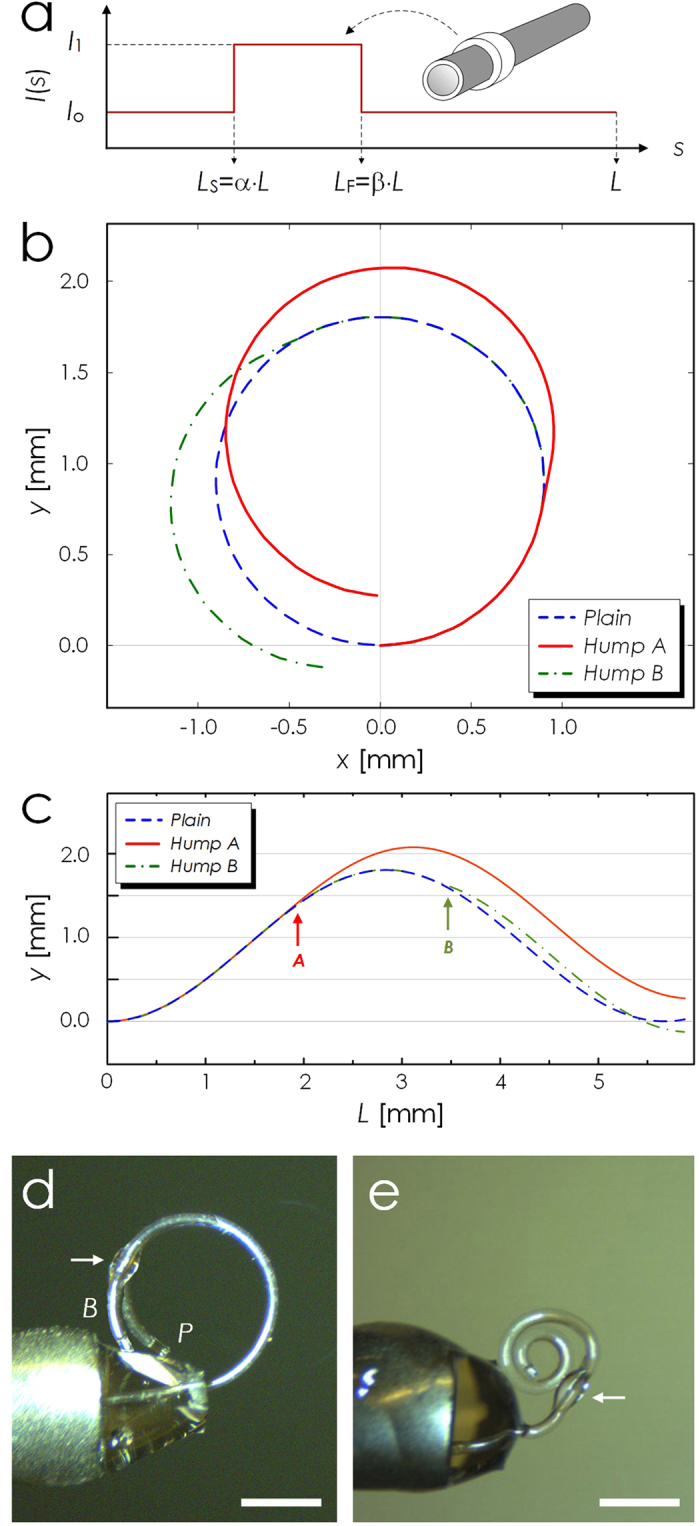
Shape-engineering of PDMS micro-tentacles. (**a**) A schematic diagram of a humped PDMS micro-tentacle and the change in its *I* due to the hump. Calculated loci (**b**) and *y*-values (**c**) of three microtubes with different hump configurations. *Plain*: no axial change in cross-sectional shape, *Hump A*: with a properly positioned hump (*L*_s_ = 0.21·*L*), and *Hump B*: with an improperly positioned hump (*L*_s_ = 0.55·*L*). *Hump A* re-entered the first quadrant with *y* > 0, achieving an inward spiraling. *Hump B*, on the other hand, actually rendered the bending worse than that of the non-humped *Plain*. (**d**) Overlapped images of microtube actuations before (*P*) and after (*B*) installing an improperly positioned hump (arrow-marked). The RoC got bigger even with the hump. (**e**) Micrograph of a micro-tentacle with a hump (arrow-marked) capable of achieving a 2-turn spiraling with the final RoC of 210 μm. (Scale bars: 1 mm).

We investigated the hump’s role in promoting the spiraling motion using the Euler-Bernoulli beam theory^31^,^41^,^42^ At the instant when the microtube nearly forms a ring shape, the coordinates (*x*, *y*) of the deformed base curve can be described by an integration:
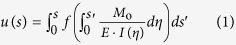
where *s* ∈ [0, *L*] is the natural coordinate of the beam of length *L*, and *f* is a cosine (sine) function for *u* *=* *x* (*u* *=* *y*). *E* is the material’s Young’s modulus, and *I* is the second moment of area. At this specific instant, it is plausible to assume that a plain microtube has a constant *I* along its length, and that a humped microtube has a step-wise distribution of *I*, as shown in Fig. 3a. Herein, we restrict our qualitative investigation to elastic regime at an instant with a fixed pressure. The full evolution of *I* with increasing pressure would call for analyses of hyperelastic deformations and plasticity at extreme cases, which is beyond our scope. The bending moment *M*_o_ ≡ π·*r*^2^·*p*·*d*_e_ wher_e_ *r* is the radius, *p* the current pressure, and *d*_e_ the *d*istance between the microtube's neutral axis and void hole's center. Figure 3b shows that the plain microtube will bend gradually to form a ring but will not achieve spiraling. It is clear from the plot that an inward spiraling requires re-entry of the end-point into the first quadrant with its *y* > 0. In light of the simple harmonic functions in equation (1), the integration over a full cycle will reset both *x* and *y* to 0, thereby impeding the desired spiraling.

With the hump, we can modulate the microtube’s geometry and, hence, the values of *I* as shown in Fig.3a. Considering the step-wise distribution of *I*, the integration in equation (1) will be split into three, each covering the pre-hump, hump, and post-hump sections as:
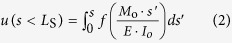

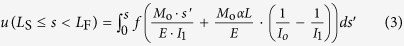

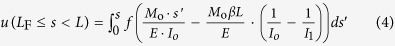
where *I*_1_ and *I*_0_ are the values of *I* in the hump and non-hump sections, respectively, as shown in Fig. 3a. The position and length of the hump are also specified through α and β in Fig. 3a. Equations (2, 3, 4) and Supplementary Fig. S1 shows that the sinusoidal functions in the integrands will obtain abrupt phase shifts when *I*_0_ < *I*_1_. By adjusting the levels of the phase-shifts in *x* and *y*, we can control re-entry point to the first quadrant. A simple criterion can be derived by assuming 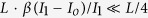. In that case, spiraling can be ensured with 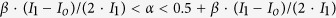.

Figures 3b and c show how a hump (length = 0.05·*L*) affects the level of bending as a function of its position. We used a microtube model with its characteristics approximately matching those of MT1. With *I*_0_ and *I*_1_ at 5.1 × 10^−16^ and 8.6 × 10^−15^ m^4^, respectively, the inward spiraling can be obtained when 0.024 < α < 0.524. We set the α value of *Hump B* at an improper value of 0.55 while setting that of *Hump A* to the proper value of 0.21, as indicated by arrows in Fig. 3c. Despite their identical size and shape, the two humps impacted the microtube’s bending very differently. As predicted, *Hump A* did achieve inward spiraling with the final *y* > 0. *Hump B*, on the other hand, performed even worse than the plain, un-humped microtube, resulting in *y* < 0.

To experimentally confirm the prediction above, we fabricated another PDMS microtube with structural characteristics very similar to those of MT1 with *L*, *d*_i_, *t*_1_, and *t*_2_ at 5800, 104, 33, and 7.4 μm, respectively, and compared its bending behaviors before and after adding a hump at an obviously improper position of 0.78·*L*. Figure 3d shows the results. In good agreement with the theoretical prediction, the improperly positioned hump resulted in bending radius even greater than that of its non-humped prototype.

Figure 3e, on the other hand, shows one of the best spiraling results observed so far. As shown in the optical micrograph, the optimized microtube actuator, with *L*, *d*_i_, *t*_1_, and *t*_2_ at 5470, 105, 34.6, and 8.2 μm, respectively, and its hump installed at 0.17·*L* point exhibited spiraling with two full turns, achieving the final RoC of 210 μm. RoC as low as 185 μm has been obtained.

### Characterization of micro-tentacle actuation

**Figure 4:**
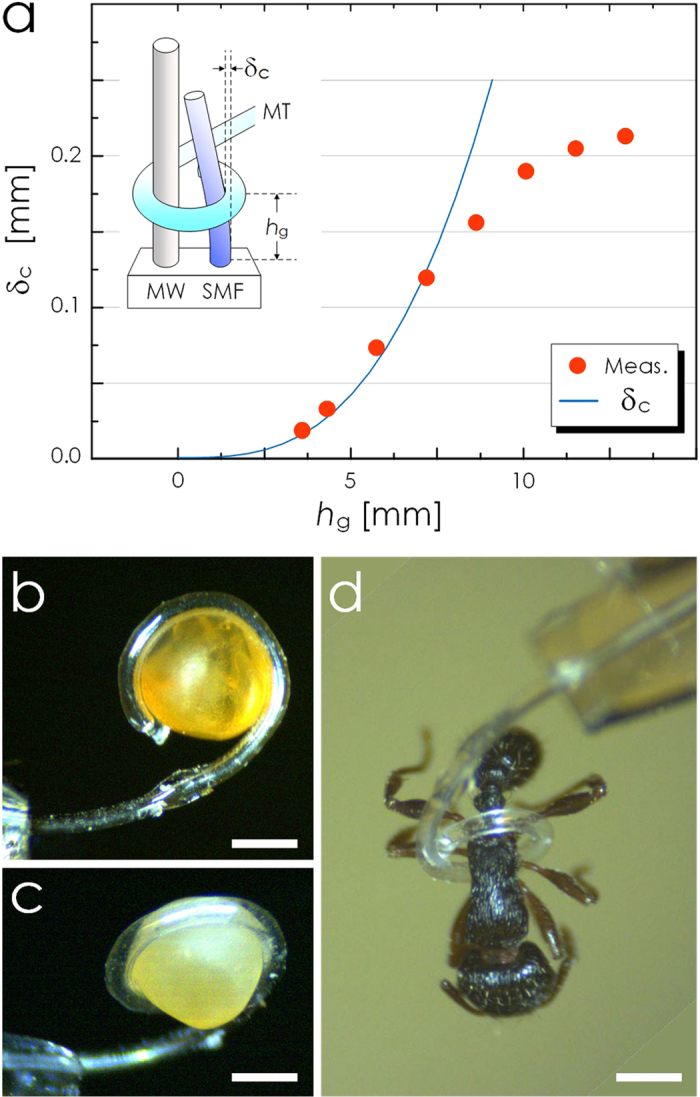
Characterization of PDMS micro-tentacle actuation. (**a**) Measured deflection of the cantilever due to the PDMS micro-tentacle’s grabbing force. The inset shows the experimental setup (MW: metal wire, SMF: glass optical fiber, MT: micro-tentacle). The solid line represents δ_c_, the deflection at the grabbing point (***h***_**g**_), calculated based on the standard beam deflection theory with the point-loaded force of 0.78 mN. The measured δ_c_ begins to deviate from the theory after it exceeds 90 μm, the maximum stroke achievable with the current setup. (**b**) and (**c**) Optical micrographs showing the micro-tentacle’s ability to grab and hold a *Mallotus villosus* egg by winding around it conformally. (**d**) Optical micrograph of another micro-tentacle grabbing and holding an ant. (Scale bar: 500 μm for all).

The spiral formed by the PDMS micro-tentacle is ideal for grabbing and holding microscale objects. To estimate its grabbing force, we configured it to deflect a cantilever as shown in the inset of Fig. 4a. As the cantilever, we used a 15 mm-long section of 125 μm-diameter fused silica optical fiber. We also installed a rigid, 155 μm-diameter metal wire in parallel with the fiber. Their surface-to-surface separation was 290 μm. Then, we wound the micro-tentacle around both the metal wire and optical fiber so that its grabbing force can function as a point load at *h*_g_ to the optical fiber cantilever. The standard beam deflection theory relates the force *F* and the deflection δ_c_ at *h*_g_ as^41^,^42^:
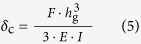
where *I* = π*r*^4^/4, *r* the radius of the optical fiber cantilever, and *E* the Young’s modulus of fused silica.

For this measurement, we used a micro-tentacle with *L*, *d*_i_, *t*_1_, and *t*_2_ at 5000, 107, 39.1, and 7.2 μm, respectively. Its minimum spiraling diameter was 370 μm and the corresponding maximum achievable deflection δ_c,max_ was 200 μm. Figure 4a shows the measured values of δ_c_ as a function of *h*_g_ at the pressure level of 9.8 psi. Each represents the average of five measurements. We omitted the error bar since the standard deviations were <1% of the average values. The force *F*, estimated through curve-fitting, was approximately 0.78 mN. We retained only the first four points in the curve-fitting since δ_c_ approached its maximum possible value and began to saturate beyond *h*_g_ ~ 7.5 mm.

Thanks to the softness of PDMS and the spiraling motion, the micro-tentacle can function as a soft-robotic grabber of micro-objects that can easily be deformed or damaged under hard material-based grippers. As practical examples, we attempted to grab biological objects. We utilized a micro-tentacle with *L*, *d*_i_, *t*_1_, and *t*_2_ at 5000, 104, 31.5, and 6.9 μm, respectively. Our first target was the egg of *Mallotus villosus* which deforms and bursts easily when manipulated with hard tweezers. We initially placed multiple eggs on a glass slide and then used the micro-tentacle’s bending motion to separate one egg. Then we grabbed the egg by winding the micro-tentacle around it. The optical micrographs in Fig. 4b and c were obtained while the egg was being held up by the micro-tentacle. In particular, Fig. 4b shows that the micro-tentacle conformally wound itself around the irregularly shaped egg, giving it minimal mechanical stress. We observed no sign of deformation in the released egg. As shown in Fig. 4d, we also grabbed and held an ant (approximately 400 μm across the waist) without damaging its body.

## Discussion

The cantilever deflection measurement revealed that the spiraling micro-tentacle’s grabbing force is in the vicinity of 0.78 mN at 9.8 psi pneumatic pressure. In absolute sense, this force is weaker than those of existing elastomer-based pneumatic micro-actuators. For example, the PDMS/polyimide-based pneumatic balloon actuator by Konish *et al.* exhibited 10−50 mN of force^43^ Smaller elastomeric micro-manipulators reported by Watanabe *et al.* and Wakimoto *et al.* also exhibited force levels of 3 mN^11^ and 2.2 mN^12^, respectively. When dealing with microscale actuators, however, their size must be taken into consideration as well. The force-volume ratios for the three actuators above are 0.78, 3.3, and 0.047 mN/mm^3^, respectively. The force-volume ratio of our micro-tentacle is 8.4 mN/mm^3^ which is higher or comparable to those listed above. It indicates that our micro-tentacle produced force very efficiently with a simple and small structure. In fact, our micro-tentacle can be regarded as the bridge between the conventional, larger micro-actuators producing mN-level force and biological micro-organisms such as *C. elegans* capable of producing 62 μN of force^44^ Given that multi-cell aggregates exhibit resilience against force at least up to 1 mN^13^, the sub-mN force level of the micro-tentacle can be highly useful for biomedical cellular manipulation.

To conclude, we have demonstrated elastomer-based soft-robotic micro-tentacles capable of winding around and holding microscale objects. To realize the thin, highly deformable microtubes, we established a new fabrication technique based on *in situ* thermal solidification of PDMS dip-coated around a cylindrical template and direct peeling of the cured structure. Its capability to asymmetrize the microtube’s cross-sectional shape enabled the microtube to bend up to a single turn. But we went further to amplify the bending into a life-like, multi-turn spiraling motion. To that end, we established a semi-analytical model to shape-engineer the microtube and turn it into a micro-tentacle. The optimized micro-tentacle exhibited a spiraling motion with two full turns and ~200 μm inner radius, which is ideal for grabbing micro-objects.

Experimental confirmation of the feasibility of such a winding motion in elastomer-based microscale pneumatic actuators is another of this work’s contribution. The spiraling capability will render the micro-tentacle particularly useful for manipulating fragile or easily deformable objects since it will allow the micro-tentacle to grab and hold a delicate object either by winding around it conformally or by forming a ring that can scoop up the object without squeezing. Thanks to the use of PDMS for its construction and also to its microorganism-level force, our micro-tentacle is fully compliance-matched to biological structures^2^ and will be ideal for future *in vivo* biomedical manipulation or surgery^7^,^8^ and endovascular operations^14^,^45^ where tissue safety holds the highest priority.

## Methods

### Microtube fabrication

As depicted in Fig. 1b, we started out with the preparation of the cylindrical template. Sodium dodecyl sulfate (SDS, Fisher Scientific), a surfactant, was mixed with water at 1:10 weight ratio. Then we treated a cylindrical template with the surfactant solution by dipping it into the mixture for 10 minutes. We used short sections of ~100 μm-diameter polyamide wire (Stroft ABR, Stroft) and 125 μm-diameter fused silica optical fiber (SMF-28, Corning) as the cylindrical template. The former turned out to be better in realizing multi-turn spiraling micro-tentacles with smaller final RoCs when compared with the latter. In parallel, we prepared a thin layer of PDMS by dispensing a drop of liquid-phase PDMS into a rectangular, 167 μm-deep recess on a glass substrate and removing the excess PDMS with a miniature squeegee. Then, we pre-baked the PDMS thin film with a hot plate set at 70 °C for 8 minutes and left it at room temperature for 30 minutes. Upon the completion of pre-baking, we immersed the surfactant-treated template into the PDMS thin film and dip-coated it with PDMS. The PDMS-coated template was then post-baked at 130 °C for 10 minutes. During the post-bake, gravity induced the PDMS under baking to flow downward, giving eccentricity to the cross-sectional shape of the PDMS coating. After the post-bake, we peeled the PDMS layer off the template using a standard optical fiber jacket remover (F-STR-103D, Newport). We then placed the completed eccentric PDMS microtube on a pre-made PDMS block, poured liquid-phase PDMS, and then cured the whole assembly on a hot plate set at 130 °C for 10 minutes. We also sealed the open-end of the microtube by dispensing a PDMS droplet. Finally, we dispensed a 35 nL PDMS droplet on the exterior of the microtube to realize the hump structure.

### Grabbing force measurement

For grabbing force measurement, we utilized a 15 mm-long section of 125 μm-diameter communication-grade fused silica optical fiber (Corning, SMF-28) as the cantilever. As the support for applying the grabbing force, we also installed a 155 μm-diameter stainless steel wire (Small Parts) in parallel with the fiber. Then we measured the deflection of the fiber under an optical microscope (E-Zoom6, Edmund Optics) as the micro-tentacle wound around both the metal wire and optical fiber.

## Additional Information

**How to cite this article**: Paek, J. *et al*. Microrobotic tentacles with spiral bending capability based on shape-engineered elastomeric microtubes. *Sci. Rep.* **5**, 10768; doi: 10.1038/srep10768 (2015).

## Supplementary Material

Supplementary Information
