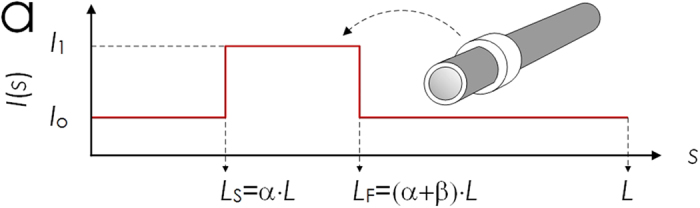# Corrigendum: Microrobotic tentacles with spiral bending capability based on shape-engineered elastomeric microtubes

**DOI:** 10.1038/srep14151

**Published:** 2015-09-21

**Authors:** Jungwook Paek, Inho Cho, Jaeyoun Kim

Scientific Reports 5: Article number: 1076810.1038/srep10768; published online: 06112015; updated: 09212015

This Article contains a typographical error in Fig. 3a, where ‘*L*_F_ = β·*L*’ should read ‘*L*_F _= (α + β) · *L*’. The correct Fig. 3a appears below as Fig. 1.

## Figures and Tables

**Figure 1:**