# Supplementary material for: Microrobotic tentacles with spiral bending capability based on shape-engineered elastomeric microtubes
Source: Sci Rep. 2015 Jun 11;5:10768. doi: 10.1038/srep10768 (PMC4463937; doi:10.1038/srep10768)
Supplement: Supplementary Information [file srep10768-s1.pdf]

Microrobotic tentacles with spiral bending capability based on shape-engineered elastomeric microtubes

Jungwook Paek[1], Inho Cho[2], and Jaeyoun Kim[1]

[1]Department of Electrical and Computer Engineering
Iowa State University, Ames, Iowa, USA

[2]Department of Civil, Construction and Environmental Engineering
Iowa State University, Ames, Iowa, USA

Supplementary Information

Supplementary Figures

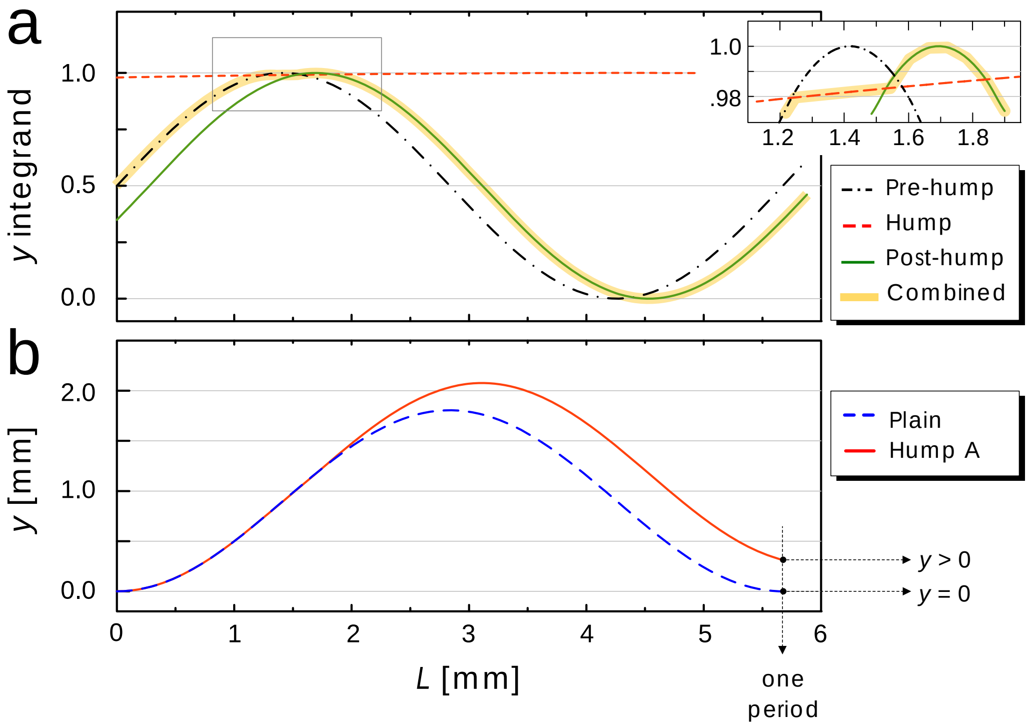

Figure S1 Changes in the integrands due to the hump

(a) The changes in the *y*-integrands for the pre-hump, hump, and post-hump sections specified in equations (2)-(4), respectively. For the humped microtube, the integration must be performed along the highlighted "combined" route, *i.e.*, first along the pre-hump curve (black, dash-dot), then the hump curve (red, dashed), and then the post-hump curve (green, solid). For the un-humped one, it suffices to integrate along the pre-hump curve. (b) The integration results for the plain, un-humped microtube (blue, dashed) and the humped one (red, solid). For the integration over a full cycle, the purely sinusoidal integrand of the former ended up $y = 0$. In contrast, with $l_1 >> l_o$, the hump section curve changes much more slowly than those in other sections and can function as a phase-shift between them (as shown in the inset in detail), eventually altering the integration result to a non-zero, positive value. This enables the inward spiraling of the micro-tentacle.
